# Design of a synthetic luminescent probe from a biomolecule binding domain: selective detection of AU-rich mRNA sequences†
†Electronic supplementary information (ESI) available: Details of synthetic procedures of LTIS^Tb^, recombinant expression of TTP-2D and spectroscopic characterization of LTIS^Tb^ and its RNA-binding properties. See DOI: 10.1039/c6sc04086a



**DOI:** 10.1039/c6sc04086a

**Published:** 2016-11-16

**Authors:** Laurent Raibaut, William Vasseur, Geoffrey D. Shimberg, Christine Saint-Pierre, Jean-Luc Ravanat, Sarah L. J. Michel, Olivier Sénèque

**Affiliations:** a Univ. Grenoble Alpes , LCBM/PMB , F-38000 Grenoble , France; b CNRS , LCBM/PMB , UMR 5249 , F-38000 Grenoble , France; c CEA , BIG-CBM , PMB , F-38000 Grenoble , France . Email: olivier.seneque@cea.fr; d Department of Pharmaceutical Sciences , School of Pharmacy , University of Maryland , Baltimore , Maryland 21201-1180 , USA . Email: smichel@rx.umaryland.edu; e Univ. Grenoble Alpes , INAC-SyMMES , F-38000 Grenoble , France; f CEA , INAC-SyMMES , F-38000 Grenoble , France

## Abstract

We report the design of a luminescent sensor based upon the zinc finger (ZF) protein TIS11d, that allows for the selective time-resolved detection of the UUAUUUAUU sequence of the 3′-untranslated region of messenger RNA. This sensor is composed of the tandem ZF RNA binding domain of TIS11d functionalized with a luminescent Tb^3+^ complex on one of the ZFs and a sensitizing antenna on the other. This work provides the proof of principle that an RNA binding protein can be re-engineered as an RNA sensor and, more generally, that tunable synthetic luminescent probes for biomolecules can be obtained by modifying biomolecule-binding domains.

## Abstract 2

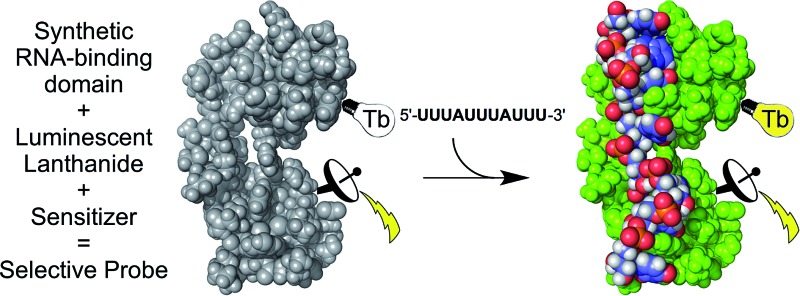
We report the design of a luminescent sensor based upon the zinc finger protein TIS11d, that allows for the selective time-resolved detection of the UUAUUUAUU sequence of the 3′-untranslated region of messenger RNA.

## Introduction

Fluorescence spectroscopy is routinely utilized in laboratories to study biological systems because it is a sensitive, cheap and easy-to-use technique.^1^ To go one step further in understanding life at the molecular level, smart luminescent probes that selectively detect, quantify or image the key components (DNA, RNA, protein, small molecules or metal cations) involved in specific biological processes are needed. Even though synthetic chemists have developed a large number of low-molecular-weight fluorescent probes that can detect various analytes (reactive oxygen species, amino acids, anions or metal cations) or that can respond to their microenvironment (pH, redox potential),^2–8^ selective sensing of larger biomolecules such as proteins, DNA or RNA is more challenging with small molecular probes.^4,9–16^ Compared to low-molecular-weight analytes, the selective recognition of large biomolecules requires a high number of interactions between the probe and its target, which are difficult to implement and control with small synthetic molecular probes.

An attractive strategy for the design of luminescent probes of biomolecules is to exploit the native recognition properties of certain biomolecules for other biomolecules. For instance, DNA/RNA or DNA/DNA recognition has been used successfully to design selective fluorescent probes for specific RNA or DNA sequences. The approach relies on the hybridization of the target RNA or DNA strand with a complementary DNA strand‡
‡The complementary strand may be a DNA oligonucleotide but alternate oligonucleotides such as locked nucleic acid (LNA) or peptide nucleic acid (PNA) can be used to create the probe strand. that incorporates a suitable fluorophore (and a quencher in the case of molecular beacons).^10,12,17,18^ The binding domains of proteins are also attractive for the design of probes for various analytes, including biomolecules. Here, the best examples are the genetically encoded probes that have been developed for various small bio-analytes,^19–24^ (metal cations (Ca^2+^, Zn^2+^, Cu^+^), Cl^–^, H_2_O_2_, cAMP) or larger biomolecules like DNA^25^ or RNA^26^ oligonucleotides. These probes were created by linking fluorescent proteins with a protein that binds the analyte of interest. One advantage of this approach is that the binding domains of proteins intrinsically feature the required selectivity and affinity as well as association and dissociation kinetics in a suitable range for reversible and dynamic exchange of the analyte in biological media. This makes them ideal recognition units to design probes for biological applications. However, there are several drawbacks to the use of genetically encoded sensors: they can only be used in organisms that can be genetically modified and control of the levels of the probe's expression, localization and optical properties is not straightforward. Alternative strategies, such as the development of synthetic luminescent probes of large biomolecules – and more generally bio-analytes – that can be tuned and utilized for applications *in vitro* or *in vivo* (in any organism) are needed. To meet this need, one approach would be to prepare synthetic probes that replicate the biomolecule-binding domains of proteins. However, these domains are typically large (>50 amino acids) and the entire domain must be synthesized in order for it to adopt the stable three-dimensional structure required for an optimal interaction with the target biomolecule. This approach is therefore synthetically challenging. A few examples of synthetic luminescent probes of biomolecules have been described based upon short peptide segments (<25–30 amino acids) derived from protein- or DNA- or RNA-binding proteins.^27–30^ These peptide-based probes are almost unfolded in the absence of the analyte and fold upon analyte binding. The energy cost associated with this folding event lowers the affinity of the probe for the analyte compared to the well-folded parent protein. Therefore, such probes are less sensitive. Recent progress in chemical protein synthesis^31–33^ allowed us to envision the design of synthetic luminescent probes based on biomolecule-binding domains of proteins comprising up to more than 100 amino acids and incorporating chromophores/fluorophores with optimized and tunable optical properties. Such probes should have superior sensitivity to short peptide based probes.

The main challenge in designing a luminescent sensor is to couple the binding event to the emission event, *i.e.* the change in light emission is due only to the binding of the receptor to its target. Trivalent lanthanide ions (Ln^3+^) are appealing emitting moieties for biological sensing applications because of their desirable luminescence properties.^34–37^ These properties include narrow emission bands that are not influenced by the environment, emission spectra covering the visible to NIR domain depending on Ln^3+^ and long luminescence lifetimes that allow for time-resolved detection to suppress background fluorescence contributions. Additionally, sensitization of Ln^3+^ luminescence requires the presence of a proximal chromophore, called an antenna. Once excited, the antenna transfers its energy to generate the excited state of the Ln^3+^ ion. Modulation of the lanthanide emission can be achieved by varying the antenna/Ln^3+^ distance, which alters the energy transfer.^34^ This approach has the potential to be generalizable as it only requires that the probe adopts different conformations in the bound and unbound states, affecting the antenna/Ln^3+^ distance.^34^

In this article, we demonstrate that a biomolecule-binding domain of a protein can be re-engineered into a lanthanide-based turn-on luminescent probe that retains the intrinsic binding properties of the native protein. We describe the rational design, the synthesis and the characterization of a luminescent probe that detects the 9-nucleotide long UUAUUUAUU RNA sequence located in the adenylate-uracylate-rich elements (AU-rich elements) of the 3′-untranslated region of messenger RNA (mRNA). The probe utilizes lanthanide luminescence and it was designed based upon the tandem zinc finger RNA binding domain (RBD) of TIS11d (also called ZP36L2), a protein of the tristetraprolin (TTP, also called ZP36) family that plays critical roles in mRNA regulation by simultaneously regulating multiple cytokines associated with inflammation (*e.g.* TNFα) and also functions as a tumor suppressor.^38–40^ The probe was obtained by chemical synthesis, is selective for the target mRNA sequence and exhibits a similar binding affinity compared to that of the native TIS11d RBD with the same RNA target. These findings provide the proof-of-principle that fully synthetic biomolecule-based luminescent probes that are selective for a specific target can be prepared.

## Results and discussion

### Design of the probe

**Fig. 1:**
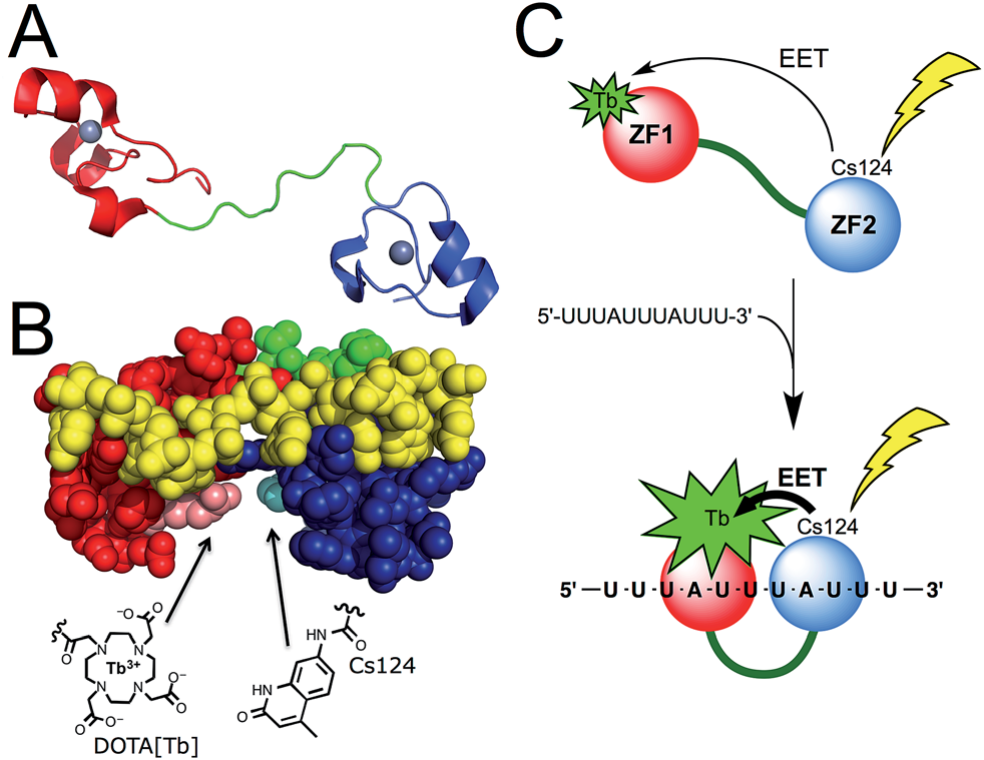
(A) Structure of TIS11d. The two zinc fingers (ZF1 and ZF2) are displayed in red and blue (with Zn^2+^ in grey) and the linker region in green. (B) NMR solution structure (pdb ; 1RGO) of TIS11d bound to the RNA nonamer UUAUUUAUU (yellow) with Arg160 and Pro210 displayed in pink and cyan, respectively.^42^ Another view of the protein is presented in Fig. S1 of ESI.† (C) Principle of AU-rich elements sensing by LTIS^Tb^.

Among the proteins of the TTP family, TTP/ZFP36, TIS11b/ZFP36L1, and TIS11d/ZFP36L2 are highly homologous and all bind to same AU-rich mRNA targets. We chose TIS11d as the basis for our designed luminescent probe because it is the only homolog for which there is an NMR structure of the two CCCH domains bound to RNA. This allowed us to identify amino acids that we could mutate without affecting the RNA binding activity. TIS11d is a *ca.* 500 amino acid protein that features a 70 amino acid RBD comprised of two non-classical CCCH zinc fingers^39,41^ separated by a *ca.* 10-residue flexible linker (Fig. 1A).^42,43^ RNA binding brings the two zinc fingers closer together and the RNA-bound TIS11d RBD (Fig. 1B) adopts a more compact fold than the free form, as revealed by its NMR solution structure and by molecular dynamic calculations.^42–44^ We speculated that this conformational change could be harnessed to design a luminescent probe, named LTIS^Tb^, by grafting a DOTA[Tb^3+^] complex onto one of the zinc fingers of the RBD and a sensitizing antenna onto the other. A more efficient electronic energy transfer (EET) between the antenna and the lanthanide was expected in the presence of the target UUAUUUAUU RNA sequence, resulting in a stronger Tb^3+^ emission (Fig. 1C). The use of an energy transfer system with an organic donor/Ln^3+^ acceptor pair rather than an organic donor/organic acceptor pair was motivated by both the luminescence properties of lanthanides and by the requirement of a short distance between the antenna and the lanthanide (below *ca.* 10–15 Å) for efficient sensitization of Ln^3+^ luminescence. Purely organic systems require distances up to 40–60 Å in order for efficient energy transfer to occur. This distance exceeds the size of the TIS11d RBD (*ca.* 40 Å, based upon the NMR structure of RNA-bound TIS11d RBD^42^).

To design LTIS^Tb^, we used a 68 amino acid peptide with the sequence corresponding to residues 151 to 218 of human TIS11d, which is the RBD.§
§In order maximize the conformational changes arising upon RNA binding, we decided to exclude Asp219 and Glu220 that were proposed to limit the unfolding of the linker region in the absence of RNA (see ref. 16). For the antenna, we utilized Carbostyril 124 (Cs124) because it is excited in the 325–350 nm which is a wavelength range where RNA or DNA nucleobases do not absorb. Cs124 is an efficient sensitizer of Tb^3+^ luminescence^45^ that can be used in the vicinity of an oligonucleotide,^46^ without quenching the sensitizing process by nucleobases.^29,47^ Due to the short antenna/Ln^3+^ distance required for efficient energy transfer, we searched for ideal positions to introduce the Ln^3+^ complex and the antenna on the TIS11d RBD sequence. The amino acid residues had to be (i) in close proximity when TIS11d RBD binds RNA, (ii) solvent exposed, (iii) not conserved among TIS11d homologs so as not to be functionally important and (iv) not involved in hydrogen bonding or hydrophobic interactions with the RNA strand or with other amino acids of the protein. Inspection of the solution structure of TIS11d^42^ revealed that Arg160 and Pro210 fulfilled all of these criteria, with a *ca.* 5.5 Å gap between their side chains.¶
¶Gly^209^, just neighbouring Pro^210^, seemed an interesting candidate but it is conserved in many tristetraprolin homologs. Therefore, positions 160 and 210 in the sequence of TIS11d were chosen to incorporate the Tb^3+^ complex and the Cs124 antenna, respectively (Fig. 1B and S1 of ESI†).

### Synthesis of the probe

**Scheme 1:**
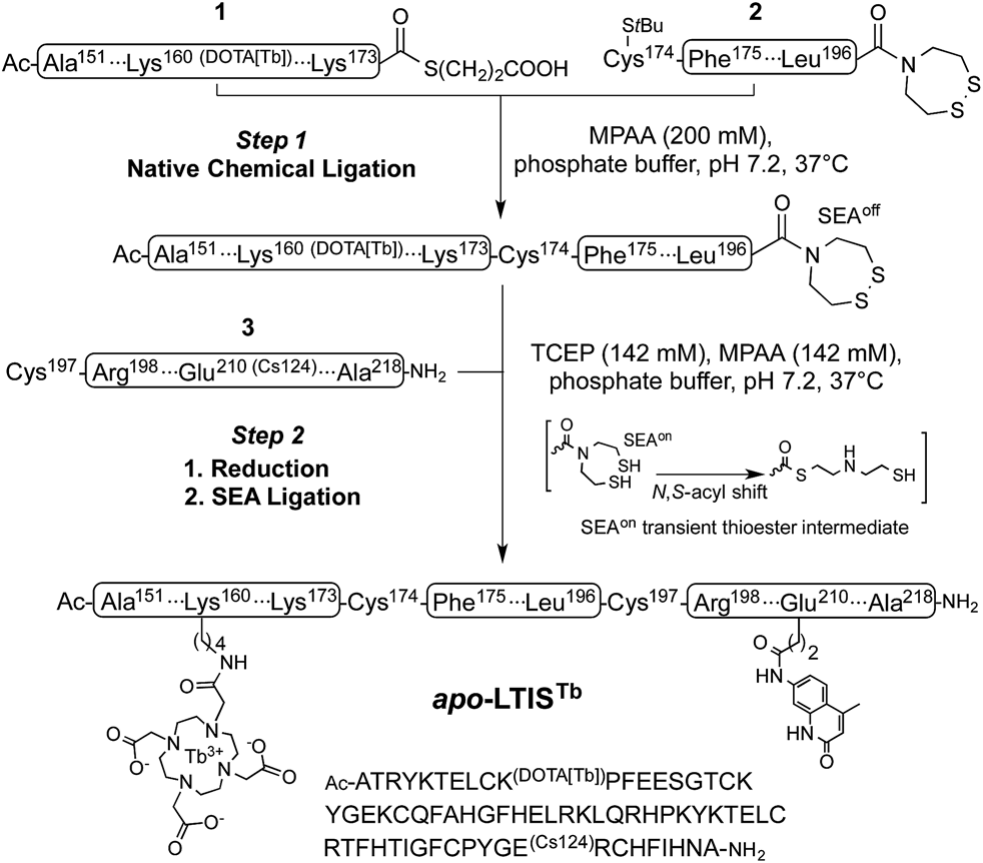
One-pot N-to-C three-segment assembly of apo-LTIS^Tb^. Amino acid numbering of TIS11d is used for clarity. The S*t*Bu group on the N-terminal Cys of segment **2** is introduced as a protecting group for synthetic reasons and is removed *in situ* by MPAA in step 1 (ESI†).

**Fig. 2:**
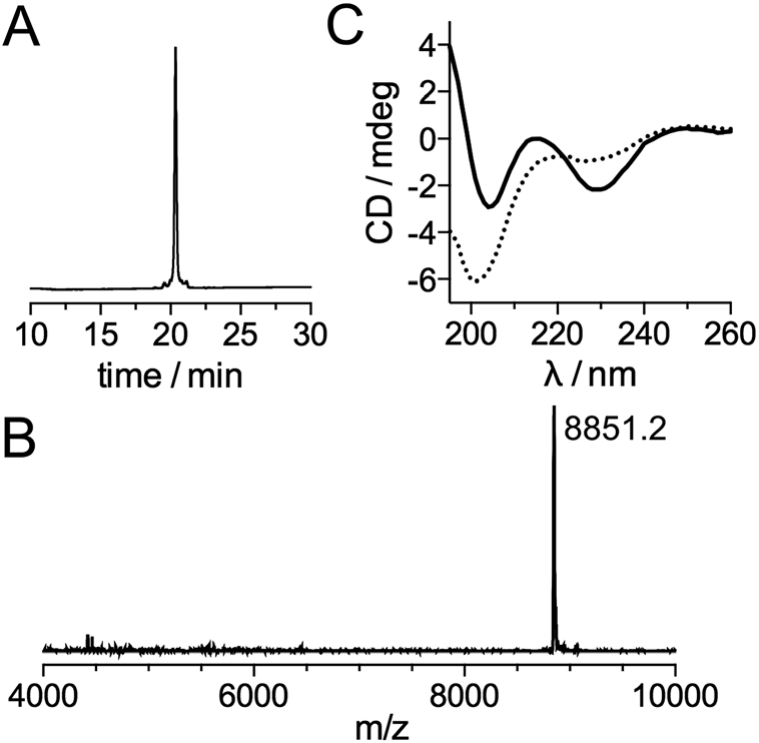
(A) HPLC chromatogram and (B) MALDI-TOF spectrum of purified apo-LTIS^Tb^ (expected MW = 8850.8). (C) CD spectra of the apo- (dotted line) and Zn-loaded (solid line) forms of LTIS^Tb^ (5 μM) recorded in a phosphate buffer (10 mM, pH 7.0).

Inspired by recent achievements in the field of protein chemical synthesis for accessing small functional protein domains,^33^ we synthesized LTIS^Tb^ by assembling three short unprotected peptide segments in water by the combination of native chemical ligation (NCL)^48^ and SEA ligation (SEA = bis(sulfanylethyl)amido),^49^ taking advantage of the numerous zinc-binding cysteines present in TIS11d RBD (Scheme 1). The N-terminal segment **1** features a DOTA[Tb] complex on a lysine side chain in the middle of its sequence and an alkylthioester at its C-end. The peptide was obtained by solid phase peptide synthesis on SEA-PS resin and the DOTA ligand was introduced onto the resin by coupling the commercially available DOTA–tris(*t*Bu) ester on the lysine side chain after removal of its alloc protecting group. After cleavage from the resin, the unprotected peptide displaying a SEA group at its C-terminus (**1a**, see ESI†) was both converted into an alkyl thioester using 3-mercaptopropionic acid and metallated with Tb^3+^ in a one-pot reaction in water at pH 4.3. The central segment **2** displays the latent thioester SEA group in its oxidized form (SEA^off^) at its C-terminus to prevent oligomerization or cyclization during NCL assembly of segments **1** and **2**.^50^ Finally, the C-terminal segment **3** displays the Cs124 chromophore on the side chain of a glutamate. This chromophore was introduced using Fmoc–Glu(Cs124)–OH^51^ during solid phase peptide synthesis of the segment. The one-pot assembly of the three segments was performed as described by Melnyk *et al.* ^50^ **1** and **2** were assembled in a phosphate buffer (pH 7.2) containing mercaptophenylacetic acid (MPAA) to unmask the C-terminal cysteine of **2** and catalyze NCL. After completion of this first ligation step (24 h), **3** was added to the reaction mixture along with the reducing agent tris(carboxyethyl)phosphine (TCEP) to convert the SEA^off^ group at the C-end of the (**1 + 2**) segment into an active SEA^on^ form and initiate the SEA ligation step.^50^ The overall process was completed within 48 h and apo-LTIS was obtained in 48% yield after purification. Fig. 2A and B show the HPLC chromatogram and mass spectrum of purified apo-LTIS.

The CD spectrum of apo-LTIS^Tb^ (Fig. 2C) is characteristic of a random coil (unstructured) peptide. A CD titration with Zn^2+^ shows that the synthesized protein can bind 2 equiv. Zn^2+^ (see ESI†) to yield LTIS^Tb^,‖
‖The 2 : 1 Zn/peptide stoichiometry was also confirmed by a luminescence titration, which shows an increase of Tb^3+^ emission up to 2.0 eq. Zn^2+^ (see ESI†). which displays a CD spectrum similar to that of the Zn-loaded RBD of TIS11d homologs with a local maximum and minimum at 215 and 230 nm, respectively.^52,53^ This indicates that LTIS^Tb^ is able to adopt the correct fold in the presence of Zn^2+^.

### RNA-binding properties

**Fig. 3:**
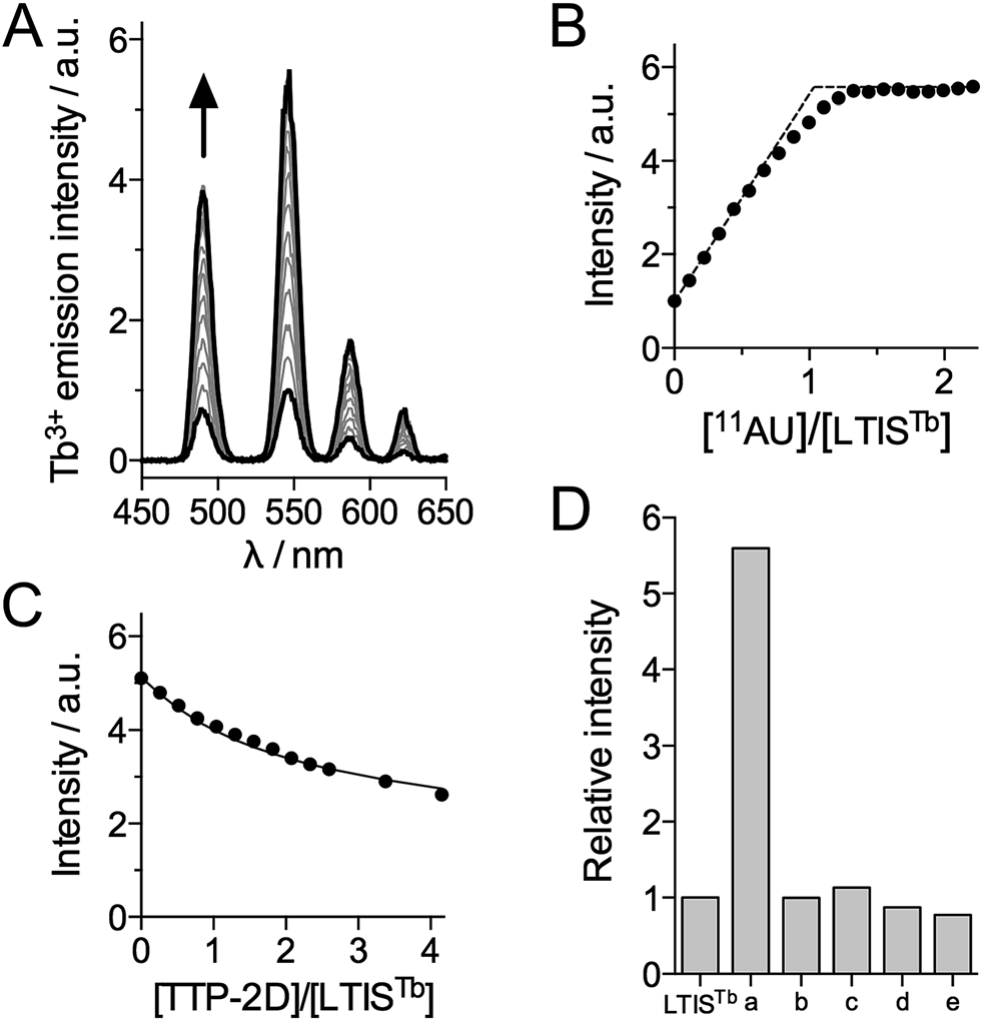
(A and B) Time-gated Tb^3+^ luminescence (*λ*_ex_ = 330 nm, delay time = 200 μs) titration of LTIS^Tb^ (90 nM) by the RNA 11-mer UUUAUUUAUUU (^11^AU). (C) Time-gated Tb^3+^ luminescence titration of a mixture of LTIS^Tb^ (90 nM) and ^11^AU RNA (90 nM) by recombinant TTP-2D. The solid line in (C) corresponds to the fit of the data, which yielded *K*_LTISTb_/*K*_TTP-2D_ = 0.35 ± 0.03. (D) Selectivity diagram showing the relative emission of Tb^3+^ at 545 nm (*λ*_ex_ = 330 nm) of LTIS^Tb^ (100 nM) in the absence and the presence of various RNA (a = ^11^AU = UUUAUUUAUUU; b = UUUUUUUUUUU; c = UUUGUUUAUUU; d = UUUGUUUGUUU) and DNA (e = TTTATTTATTT) 11-mer oligonucleotides (200 nM). Solutions were prepared in a 10 mM HEPES/50 mM NaCl/DTT 2.5 mM/0.7 mg mL^–1^ BSA buffer at pH 7.5 at 298 K.

The RNA binding properties of LTIS^Tb^ were investigated by luminescence spectroscopy in HEPES buffer (pH 7.5) with the RNA 11-mer UUUAUUUAUUU (^11^AU), which comprises the target sequence of proteins of the TTP family. In the absence of RNA, excitation at 330 nm promotes emission from the ^5^D_4_ excited state of Tb^3+^ with characteristic ^5^D_4_ → ^7^F_*J*_ (*J* = 6, 5, 4, 3) transitions at 490, 545, 585 and 623 nm, respectively (Fig. 3A). The excitation spectrum confirms that Cs124 acts as a sensitizing antenna for Tb^3+^ luminescence (see ESI†). Upon addition of ^11^AU to LTIS^Tb^ (90 nM), the intensity of the Tb^3+^ emission increases progressively and plateaus after 1 equiv. RNA, with a 5.6-fold enhancement of Tb^3+^ emission (Fig. 3A and B). This indicates the formation of a high affinity 1 : 1 complex ^11^AU·LTIS^Tb^ and confirms that LTIS^Tb^ acts as a turn-on sensor for the target RNA sequence, as expected. The Tb^3+^ luminescence lifetime remains constant and equal to 1.9 ms during the titration.

In order to assess the dissociation constant *K*_d_ of the ^11^AU·LTIS^Tb^ complex and assess the reversibility of RNA binding, we performed a competition titration with TTP-2D, a recombinant protein corresponding to the RBD of murine TTP protein, which has a *K*_d_ of 16 ± 1 nM for ^11^AU.^54^ As expected, addition of TTP-2D to a 1 : 1 mixture of LTIS^Tb^ and ^11^AU induces a decrease of Tb^3+^ emission in agreement with the displacement of ^11^AU from LTIS^Tb^ to TTP-2D (Fig. 3C). Fitting the data to the equilibrium ^11^AU·LTIS^Tb^ + TTP-2D ⇌ LTIS^Tb^ + ^11^AU·TTP-2D yielded a *K*_d_ of 5.6 ± 0.8 nM for ^11^AU·LTIS^Tb^, in the 3–7 nM range reported for TIS11d RBD.^43^ These data show that the introduction of the DOTA[Tb] complex and the Cs124 antenna on the RBD does not affect RNA binding properties. Of note, during all of these titrations, equilibrium is reached within the mixing time (<10 s), which indicates that the association and dissociation kinetics for ^11^AU·LTIS^Tb^ are rapid, a property that is required for efficient real time monitoring of reversible biological processes. Another critical issue for biological probes is selectivity. The selectivity of the luminescence response of LTIS^Tb^ was investigated by measuring the response of LTIS^Tb^ with the 11-mer RNA sequence in which one or the two As were changed for U or G (UUUUUUUUUUU, UUUGUUUAUUU and UUUGUUUGUUU, which are common RNA benchmarks for TTP protein selectivity^52,54^) as well as the DNA sequence TTTATTTATTT (Fig. 3D). None of these sequences promoted any Tb^3+^ luminescence increase, indicating that LTIS^Tb^ responds selectively to the target UUAUUUAUU RNA sequence, much like the native peptide.

### Discussion

We have described the successful design and *in vitro* analysis of a selective luminescent probe for the RNA sequence UUAUUUAUU. This sequence is the binding site for TTP family proteins. In cells, the interaction of TTP with these RNA sequences is a critical step in the regulation of inflammation and cancer. Thus, a probe that monitors this pathway has potential as a novel tool to study inflammation and cancer signalling pathways. For example, TTP simultaneously regulates multiple mRNAs that encode select pro-inflammatory factors (*e.g.*, TNFα, interleukins (IL)-2, -3, and -6, COX-2) and loss of TTP results in systemic hyperinflammation.^55–59^ A TTP-based probe could function to measure these cytokine levels in cells under different stimuli and therefore serve as a read-out of inflammation. Similarly, TTP recognizes and destabilizes a large number of tumor-related mRNAs (*e.g.* regulators of the cell cycle (*e.g.* cyclin D1), angiogenesis (*e.g.* VEGF, HIF-1), and metastasis (*e.g.* MMP-1, uPA)),^60–64^ and we envision using the TTP-based probes to monitor mRNA levels in cancer cells. Our approach involved modifying the RBD of one of the TTP family proteins (TIS11d) with a lanthanide ion (Tb^3+^) and a suitable chromophore to sensitize lanthanide luminescence. By using TIS11d as the basis for the probe, we created a probe that kept the native RBD's selective RNA binding properties, *i.e.* reversible and dynamic binding with the *K*_d_ in the nM range.

LTIS^Tb^ is a rare example of a protein-based RNA sensor.^29,30^ Most RNA sensors described to date are based on hybridization with a nucleic acid oligomer-based fluorescent probe. With such a probe, the target RNA sequence must be longer than 15 nucleotides to form an RNA/probe hybrid with sufficient stability. LTIS^Tb^ recognizes a shorter RNA sequence (UUAUUUAUU, 9 nucleotides) showing that this protein-based approach may be a valuable alternative to hybridization probes for the sensing of short RNA sequences.

In the LTIS^Tb^ probe, the RNA binding event is transduced into an increased Tb^3+^ emission based upon the modulation of Tb^3+^ sensitization though a conformational change between the Cs124 antenna and Tb^3+^ ion. The use of a lanthanide emitter is critical to the design of LTIS^Tb^ as small conformational changes are sensed due to the short distances required for efficient energy transfer from the antenna. The lanthanide complex and its antenna were introduced on the RBD of TIS11d based upon simple structural and functional considerations. Moreover, lanthanide luminescence offers unique properties to study biological systems. They have a long luminescence lifetime – exemplified here by the 1.9 ms lifetime for Tb^3+^ luminescence in LTIS^Tb^ – that allows the time-resolved detection to suppress autofluorescence of the biological background and increase the signal/noise ratio. With this promising prototype probe in hand, we envision future studies in which modifications are made to the probe for different applications. For example, we could apply the convergent synthetic pathway for LTIS^Tb^ in a combinatorial manner by varying the Ln^3+^ ion, its ligand (the DOTA ligand usually leaves a free coordination site on the lanthanide that is occupied by water molecules and this is detrimental to emission efficiency), the antenna and the position of the DOTA and antenna on the RBD to optimize the probe's emission properties. Additionally, we envision appending functionalities for *in vivo* applications, *e.g.* addition of a cell penetrating peptide sequence or a second lanthanide complex to create a ratiometric sensor.

The work we have described here demonstrates that a fully synthetic luminescent probe of a target analyte (RNA) based upon a biomolecule-binding domain of a protein can be engineered. By utilizing the robust chemical ligation methodologies that have been developed in the past decade for the chemical synthesis of proteins, the straightforward preparation of a biomolecule with a conjugated antenna and lanthanide complex from short peptide segments was achieved. The biomolecule utilized as the basis for the probe contains two zinc finger domains. These types of domains are amenable to chemical synthesis *via* chemical ligation methods^65–67^ because they contain numerous zinc-binding cysteines (2 to 4 per Zn^2+^ ion, depending on the type of zinc finger). In addition, Nature often uses zinc fingers to fold biomolecule-binding sites and mediate protein/protein, protein/DNA, protein/RNA and protein/low-molecular-weight molecules interactions, suggesting that our approach can be expanded to target other biomolecules by functionalizing other zinc fingers. Additionally, this design strategy is not limited to zinc finger proteins or cysteine-containing proteins as progress in chemical ligation methods now allows straightforward synthesis of proteins that do not contain cysteines,^33,68^ thereby extending the scope of such probe design.

## Conclusion

We have successfully engineered a luminescent sensor for the RNA sequence UUAUUUAUU by modifying the RNA binding domain of TIS11d, a member of the TTP family, such that it contains a Ln^3+^ complex and a sensitizing antenna. The sensor was designed based on simple structural considerations and synthesized by the native chemical ligation methodology, which allows straightforward introduction of the Ln^3+^ emitter and antenna. Of note, the probe retains all of the RNA binding properties of the native TIS11d RBD, validating this approach. We believe that the design strategy has the potential to be expanded to other biomolecule-binding proteins, providing a general strategy for the development of luminescent probes.

## References

[cit1] Lakowicz J. R., Principles of Fluorescence Spectroscopy, Springer-Verlag, New York, 3rd edn, 2006.

[cit2] Li X., Gao X., Shi W., Ma H. (2014). Chem. Rev..

[cit3] Hamilton G. R. C., Sahoo S. K., Kamila S., Singh N., Kaur N., Hyland B. W., Callan J. F. (2015). Chem. Soc. Rev..

[cit4] Lee M. H., Kim J. S., Sessler J. L. (2015). Chem. Soc. Rev..

[cit5] You L., Zha D., Anslyn E. V. (2015). Chem. Rev..

[cit6] Yeung M. C.-L., Yam V. W.-W. (2015). Chem. Soc. Rev..

[cit7] Lou Z., Li P., Han K. (2015). Acc. Chem. Res..

[cit8] Kaur A., Kolanowski J. L., New E. J. (2016). Angew. Chem., Int. Ed..

[cit9] Kubota R., Hamachi I. (2015). Chem. Soc. Rev..

[cit10] Yang Y., Zhao L. (2010). TrAC, Trends Anal. Chem..

[cit11] Han Y.-W., Sugiyama H., Harada Y. (2016). Biomater. Sci..

[cit12] Armitage B. A. (2011). Curr. Opin. Chem. Biol..

[cit13] Liu Y., Jun E. J., Kim G., Lee A.-R., Lee J.-H., Yoon J. (2014). Chem. Commun..

[cit14] Laguerre A., Hukezalie K., Winckler P., Katranji F., Chanteloup G., Pirrotta M., Perrier-Cornet J.-M., Wong J. M. Y., Monchaud D. (2015). J. Am. Chem. Soc..

[cit15] Sparano B. A., Koide K. (2007). J. Am. Chem. Soc..

[cit16] Krasheninina O. A., Novopashina D. S., Lomzov A. A., Venyaminova A. G. (2014). ChemBioChem.

[cit17] Juskowiak B. (2011). Anal. Bioanal. Chem..

[cit18] Hövelmann F., Seitz O. (2016). Acc. Chem. Res..

[cit19] Zhang J., Campbell R. E., Ting A. Y., Tsien R. Y. (2002). Nat. Rev. Mol. Cell Biol..

[cit20] Morris M. C. (2010). Cell Biochem. Biophys..

[cit21] Palmer A. E., Qin Y., Park J. G., McCombs J. E. (2011). Trends Biotechnol..

[cit22] Hochreiter B., Garcia A. P., Schmid J. A. (2015). Sensors.

[cit23] Hessels A. M., Merkx M. (2015). Metallomics.

[cit24] Carter K. P., Young A. M., Palmer A. E. (2014). Chem. Rev..

[cit25] Slomovic S., Collins J. J. (2015). Nat. Methods.

[cit26] Ozawa T., Natori Y., Sato M., Umezawa Y. (2007). Nat. Methods.

[cit27] Pazos E., Vazquez O., Mascareñas J. L., Vázquez M. E. (2009). Chem. Soc. Rev..

[cit28] Boyd J. A., Ensign S. A. (2005). Biochemistry.

[cit29] Penas C., Pazos E., Mascareñas J. L., Vázquez M. E. (2013). J. Am. Chem. Soc..

[cit30] Penas C., Mascareñas J. L., Vázquez M. E. (2016). Chem. Sci..

[cit31] Kent S. B. H. (2009). Chem. Soc. Rev..

[cit32] Raibaut L., Ollivier N., Melnyk O. (2012). Chem. Soc. Rev..

[cit33] Bondalapati S., Jbara M., Brik A. (2016). Nat. Chem..

[cit34] Thibon A., Pierre V. C. (2009). Anal. Bioanal. Chem..

[cit35] Bünzli J.-C. G. (2010). Chem. Rev..

[cit36] Eliseeva S. V., Bünzli J.-C. G. (2010). Chem. Soc. Rev..

[cit37] Sy M., Nonat A., Hildebrandt N., Charbonnière L. J. (2016). Chem. Commun..

[cit38] Stumpo D. J., Lai W. S., Blackshear P. J. (2010). Wiley Interdiscip. Rev.: RNA.

[cit39] Lee S. J., Michel S. L. J. (2014). Acc. Chem. Res..

[cit40] Tiedje C., Diaz-Muñoz M. D., Trulley P., Ahlfors H., Laaß K., Blackshear P. J., Turner M., Gaestel M. (2016). Nucleic Acids Res..

[cit41] Michalek J. L., Besold A. N., Michel S. L. J. (2011). Dalton Trans..

[cit42] Hudson B. P., Martinez-Yamout M. A., Dyson H. J., Wright P. E. (2004). Nat. Struct. Mol. Biol..

[cit43] Morgan B. R., Deveau L. M., Massi F. (2015). Biophys. J..

[cit44] Brewer B. Y., Ballin J. D., Fialcowitz-White E. J., Blackshear P. J., Wilson G. M. (2006). Biochemistry.

[cit45] Xiao M., Selvin P. R. (2001). J. Am. Chem. Soc..

[cit46] Li L.-L., Ge P., Selvin P. R., Lu Y. (2012). Anal. Chem..

[cit47] Ancel L., Gateau C., Lebrun C., Delangle P. (2013). Inorg. Chem..

[cit48] Dawson P. E., Muir T. W., Clarklewis I., Kent S. B. H. (1994). Science.

[cit49] Ollivier N., Dheur J., Mhidia R., Blanpain A., Melnyk O. (2010). Org. Lett..

[cit50] Ollivier N., Vicogne J., Vallin A., Drobecq H., Desmet R., El Mahdi O., Leclercq B., Goormachtigh G., Fafeur V., Melnyk O. (2012). Angew. Chem., Int. Ed..

[cit51] Reynolds A. M., Sculimbrene B. R., Imperiali B. (2008). Bioconjugate Chem..

[cit52] Michalek J. L., Lee S. J., Michel S. L. J. (2012). J. Inorg. Biochem..

[cit53] Deveau L. M., Massi F. (2016). ACS Chem. Biol..

[cit54] diTargiani R. C., Lee S. J., Wassink S., Michel S. L. J. (2006). Biochemistry.

[cit55] Sanduja S., Blanco F. F., Young L. E., Kaza V., Dixon D. A. (2012). Front. Biosci..

[cit56] Emmons J., Townley-Tilson W. H. D., Deleault K. M., Skinner S. J., Gross R. H., Whitfield M. L., Brooks S. A. (2008). RNA.

[cit57] Mukherjee N., Jacobs N. C., Hafner M., Kennington E. A., Nusbaum J. D., Tuschl T., Blackshear P. J., Ohler U. (2014). Genome Biol..

[cit58] Stoecklin G., Tenenbaum S. A., Mayo T., Chittur S. V., George A. D., Baroni T. E., Blackshear P. J., Anderson P. (2008). J. Biol. Chem..

[cit59] Taylor G. A., Carballo E., Lee D. M., Lai W. S., Thompson M. J., Patel D. D., Schenkman D. I., Gilkeson G. S., Broxmeyer H. E., Haynes B. F., Blackshear P. J. (1996). Immunity.

[cit60] Al-Souhibani N., Al-Ahmadi W., Hesketh J. E., Blackshear P. J., Khabar K. S. A. (2010). Oncogene.

[cit61] Marderosian M., Sharma A. P., Funk A. P., Vartanian R., Masri J., Jo O. D., Gera J. F. (2006). Oncogene.

[cit62] Cha H. J., Lee H. H., Chae S. W., Cho W. J., Kim Y. M., Choi H.-J., Choi D. H., Jung S. W., Min Y. J., Lee B. J., Park S. E., Park J. W. (2011). Hepato-Gastroenterology.

[cit63] Chamboredon S., Ciais D., Desroches-Castan A., Savi P., Bono F., Feige J.-J., Cherradi N. (2011). Mol. Biol. Cell.

[cit64] Brennan S. E., Kuwano Y., Alkharouf N., Blackshear P. J., Gorospe M., Wilson G. M. (2009). Cancer Res..

[cit65] Beligere G. S., Dawson P. E. (1999). Pept. Sci..

[cit66] Futaki S., Tatsuto K., Shiraishi Y., Sugiura Y. (2004). Pept. Sci..

[cit67] Fehr F., Nadler A., Brodhun F., Feussner I., Diederichsen U. (2012). ChemistryOpen.

[cit68] Maity S. K., Jbara M., Laps S., Brik A. (2016). Angew. Chem., Int. Ed..

