# Supplementary material for: Design of a synthetic luminescent probe from a biomolecule binding domain: selective detection of AU-rich mRNA sequences
Source: Chem Sci. 2016 Nov 16;8(2):1658–64. doi: 10.1039/c6sc04086a (PMC5364516; doi:10.1039/c6sc04086a)
Supplement: Supplementary file 1 [file SC-008-C6SC04086A-s001.pdf]

Electronic Supplementary Material (ESI) for Chemical Science.
This journal is © The Royal Society of Chemistry 2016

Design of a Synthetic Luminescent Probes from a Biomolecule Binding Domain: Selective Detection of AU-rich mRNA Sequences

Laurent Raibaut,[a] William Vasseur,[a] Geoffrey M. Shimberg,[b] Christine Saint-Pierre,[c] Jean-Luc Ravanat,[c] Sarah L. J. Michel,[b],* Olivier Sénèque.[a],*

[a] Univ. Grenoble Alpes, LCBM/PMB, F-38000 Grenoble, France and CNRS, LCBM/PMB, UMR 5249, F-38000 Grenoble, France and CEA, BIG/CBM/PMB, F-38000 Grenoble, France

[b] Department of Pharmaceutical Sciences, School of Pharmacy, University of Maryland, Baltimore, Maryland 21201-1180, United States

[c] Univ. Grenoble-Alpes, iNAC-SyMMES, F-38000 Grenoble, France and CEA, iNAC/SyMMEs, F-38000 Grenoble, France

E-mail: olivier.seneque@cea.fr

Supporting Information

Content

Abbreviations 2
Materials and methods 2
Peptide sequences 2
Peptide synthesis 3
Expression and purification of the two-domain TTP construct 7
Zn-loading of LTIS^{Tb} 8
RNA/LTIS^{Tb} interaction: luminescence spectroscopy 8
References 10

Abbreviations

PyBOP: (Benzotriazol-1-yloxy)tripyrrolidino-phosphonium-hexafluorophosphate; HATU: N-[(dimethylamino)-1H-1,2,3-triazolo[4,5-b]-pyridin-1-ylmethylene]-N-methylmethanaminium hexafluorophosphate N-oxide; Cs124: carbostyril 124; SEA: bis(2-sulfanylethyl)-amino; SEA-PS: bis(2-sulfanylethyl)-aminotrityl-polystyrene; DOTA: 1,4,7,10-tetraazacyclododecane-1,4,7,10-tetraacetic acid; $Pd(PPh_3)_4$: *tetrakis*(triphenylphosphine)-palladium(0); TIS: triisopropylsilane; TCEP: *tris*(2-carboxyethyl)phosphine; MPA: 3-mercaptopropionic acid; MPAA: 4-mercaptophenylacetic acid; DTT: dithiotreitol; DIEA: N,N-diisopropylethylamine; TFA: trifluoroacetic acid; MeCN: acetonitrile; DCM: dicholoromethane; Et_2O: diethylether; DMF: N,N-dimethylformamide; DMSO: dimethylsulfoxide; *t*Bu: *tert*-butyl; Trt: trityl; Fmoc: 9-fluorenylmethoxycarbonyl; Boc: tert-butyloxycarbobyl; Pbf: 2,2,4,6,7-pentameethyldihydrobenzofuran-5-sulfonyl; Alloc: allyloxycarbonyl; HPLC: high performance liquid chromatography; ESI: electrospray ionization; MALDI-TOF: Matrix-assisted laser desorption/ionization-time of flight; MS: mass spectrometry; UV-Vis: ultraviolet-visible; CD: circular dichroism.

Materials and methods

Reagents and solvents: N-α-Fmoc-protected amino acids for peptide synthesis, PyBOP and HATU coupling reagent were obtained from Novabiochem or Iris Biotech. Fmoc-Glu(Cs124)-OH was synthetized according to literature procedure.[1] SEA-PS and NovaPEG Rink Amide resin were purchased from X'prochem and Novabiochem, respectively. DOTA-*tris*(tBu) ester was purchased from CheMatech. Other reagents for peptide synthesis, solvents, buffers and metal salts were purchased from Sigma-Aldrich. HPLC-purified oligonucleotides were purchased from Eurofins Genomic, dissolved in water before use and stored at -80°C. The concentrations of oligonucleotides solutions were determined by measuring the absorption at 260 nm. All buffer or metal solutions for spectroscopic measurements were prepared with MilliQ water (Millipore). Buffer solutions were treated with Chelex 100 resin (Biorad) to remove trace metal ions.

Analyses and purifications: Analytical HPLC separations were performed on an Agilent Infinity 1200 system using Merck PurospherStar RP-18e (5 µm, 150 mm × 4.6 mm) or Merck Chromolith RP-18e (100 mm × 4.6 mm) columns at 1 and 2 mL/min, respectively. Preparative HPLC separations were performed on a VWR LaPrepΣ system using a Waters XBridge Peptide BEH130 C18 (5 µm, 150 mm × 19 mm) column. Mobile phase consisted in a gradient of solvent A (0.1% TFA in H_2O) and B (0.1% TFA in MeCN/H_2O 9:1). Eluate was monitored by electronic absorption at 214, 280 and 331 nm. ESI-MS analyses were performed on a Thermo LXQ spectrometer. MALDI-TOF mass spectra were recorded in positive mode with a Bruker Microflex spectrometer. UV-Vis absorption spectra were recorded on a Perkin-Elmer Lambda 35 spectrophotometer. Emission spectra were recorded on a Cary Eclipse spectrometer. CD spectra were recorded on an Applied Photophysics Chirascan spectropolarimeter. All optical spectrometers are equipped with a thermo-regulated cell holder.

Peptide sequences

***apo*-LTIS$^{\mathbf{Tb}}$**: Ac-ATRYKTELCK(DOTA[Tb])PFEESGTCKYGEKCQFAHGFHELRKLQRHPKYKTELCRT-FHTIGFCPYGE(Cs124)RCHFIHNA-NH_2

1a: Ac-ATRYKTELC(S*t*Bu)K(DOTA)PFEESGTC(S*t*Bu)KYGEK-SEA^{off}

1: Ac-ATRYKTELCK(DOTA[Tb])PFEESGTCKYGEK-S$(CH_2)_2$COOH

2: C(S*t*Bu)QFAHGFHELRKLQRHPKYKTEL-SEA^{off}

3: CRTFHTIGFCPYGE(Cs124)RCHFIHNA-NH_2

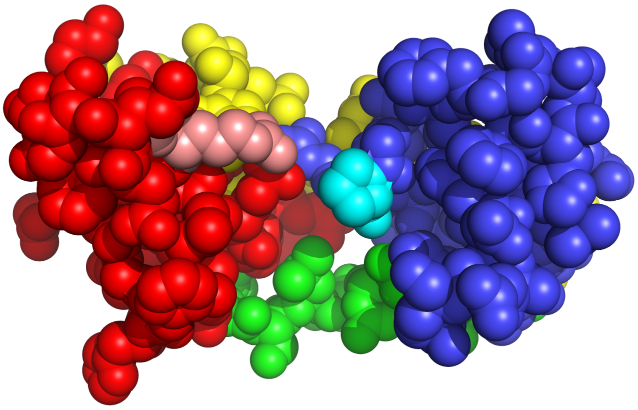

Figure S1. Alternative view of the solution structure of TIS11d in complex with RNA UUAUUUAUU (pdb 1RGO[2]) showing the positions of Arg160 (pink) and Pro210 (cyan), chosen to introduce the DOTA[Tb] complex and the Cs124 antenna. The two zinc fingers are displayed in red and blue, the linker region in green and the RNA in yellow.

Peptide synthesis

Peptide elongation: Peptide elongation was performed using standard SPPS protocols using Fmoc/*t*Bu chemistry either manually or on an automated peptide synthesizer (CEM Liberty1 Microwave Peptide Synthesizer). Double couplings (30 min) were performed using 4-fold molar excess of Fmoc-L-amino acid, 4-fold molar excess of PyBOP and 8-fold molar excess of DIEA at room temperature. A capping step was performed after each coupling with Ac_2O/DIEA in DMF (5 min). Fmoc removal was performed using 20% piperidine in DMF (2×10 min).

SEA^{off} N-terminal segment 1a: Peptide elongation was performed as described above on SEA-PS resin (0.1 mmol, 0.16 mmol/g) after attachment of the first amino acid by double manual coupling (30 min) using 10-fold excess of Fmoc-Lys(Boc)-OH, 9.5-fold excess of HATU and 10-fold excess of DIEA in DMF with pre-activation (5 min) followed by acetylation using (Ac_2O/DIEA/DCM 2:1:17 by vol., 10 mL, 2×5 min).[3] After acetylation of the N-terminus, removal of the N-Alloc protecting group of the Lys(Alloc) was performed using with $Pd(PPh_3)_4$ (0.05 mmol, 0.5 eq., 58 mg) and phenylsilane (2.5 mmol, 25 eq., 0.3 mL) in degassed anhydrous DCM (15 mL) for 1h in the dark.[4] The resin was then washed successively with DCM (2×2 min), DMF (2×2 min), 1% H_2O in DMF (2×2 min), DMF (2×2 min), 1% DIEA in DMF (2×2 min), DMF (2×2 min), sodium diethyldithiocarbamate in DMF (0.12 M, 2×5 min) and DMF (2×2 min). DOTA-tris(*t*Bu) ester (0.2 mmol, 114 mg, 2 eq.) was dissolved in a small amount of DMF and added to the resin, then a solution of PyBOP (0.2 mmol, 104 mg, 2 eq.) and DIEA (0.6 mmol, 104 µL, 6 eq) in DMF (2 mL) was added. The resin was agitated overnight at room temperature and the coupling step was repeated once for 4h. The resin was washed with DMF (2×2 min), DCM (2×2 min) and Et_2O (2×2 min) and dried. Removal of acid-labile side chain protecting groups and cleavage of the peptididyl resin was performed with TFA/H_2O/TIS/thioanisole (92.5/2.5/2.5/2.5 by vol., 10 mL) during 4h. The peptide was then precipitated in ice-cold Et_2O/heptane (1:1 by vol., 100 mL), dissolved in deionized water, and lyophilized. The peptide was dissolved in H_2O/AcOH and treated with a solution of I_2 (200 mM in DMSO) to oxidize the C-terminal SEA^{on} group into SEA^{off} group.[3,5] After 30 s, DTT (65 mM in water, 500 µl) was added to quench the excess of iodine. The oxidized peptide was immediately purified by HPLC to give **1a** (25 mg, 7% yield, 0.1 mmol scale). ESI-MS: average *m/z* = 1131.3 (3+), 848.8 (4+), 679.3 (5+) / calculated av. *m/z* = 1131.35 $[M+3H]^{3+}$, 848.76 $[M+4H]^{4+}$, 679.21 $[M+5H]^{5+}$ for M = $C_{146}H_{233}N_{35}O_{45}S_6$; deconvoluted mass found = 3391.2 / expected mass = 3391.02 (average isotopic composition).

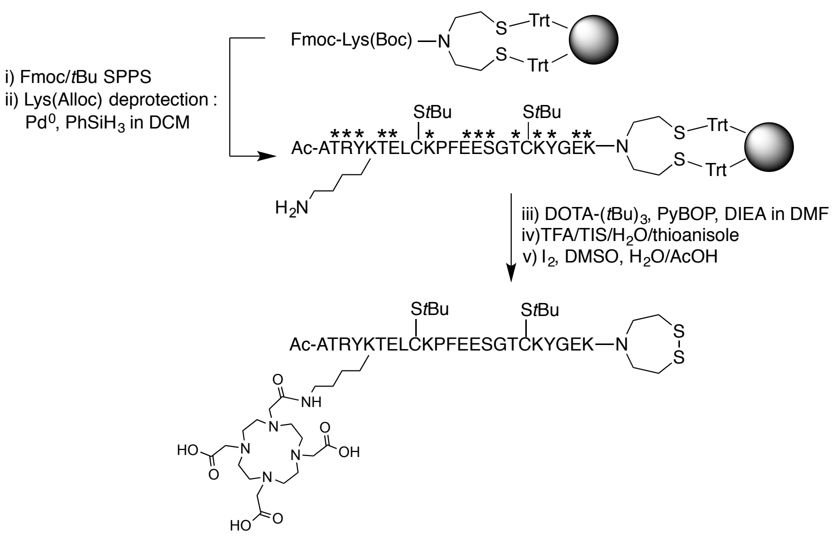

Figure S2. Synthetic pathway for preparation of the N-terminal segment **1a**. * denotes standards protecting groups for Fmoc SPPS (*t*Bu for Glu, Ser, Thr, Tyr, Pbf for Arg, Boc for Lys). The S*t*Bu protecting groups are used for the cysteines instead of classical Trityl groups to prevent irreversible oxidation of unprotected Cys residues during step (v), i.e. I_2 oxidation of the SEA group.

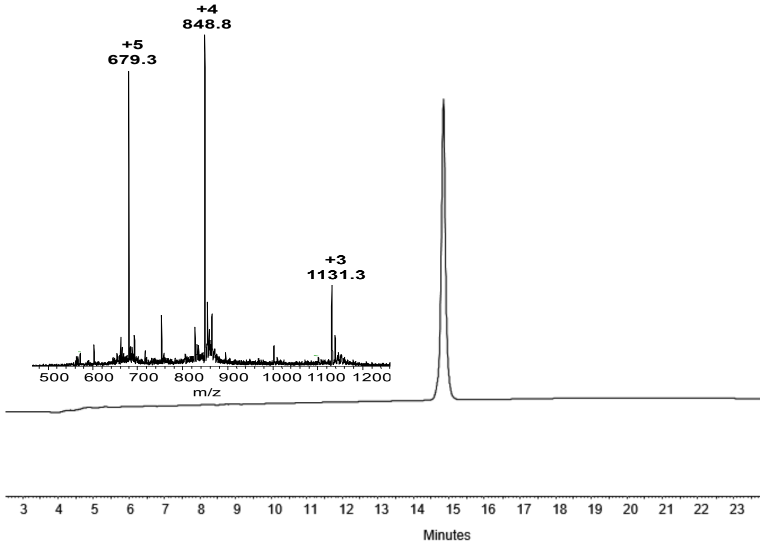

Figure S3. Analytical HPLC chromatogram (λ = 214 nm) and ESI-MS spectrum (positive mode) of the purified N-terminal segment **1a**.

N-terminal segment thioester 1: A TCEP/MPA solution was prepared by dissolving TCEP (129 mg, 0.45 mmol) in a MPA solution (5% vol. in H_2O, 5 mL) and adjusting the pH to 4.3 using aq. NaOH (1 N). **1a** (10 mg, 3 µmol) and $TbCl_3$ (13 mg, 0.035 mmol, 12 eq.) were dissolved in the above solution (4.2 mL, final peptide concentration 0.7 M). The reaction mixture was stirred at 37°C for 24 h and then diluted with water (5 mL) and 10% aqueous TFA (2 mL). The solution was extracted with Et_2O and immediately purified by HPLC to give **1** (4 mg, 40% yield). ESI-MS: average *m/z* = 1114.7 (3+), 836.3 (4+), 669.4 (5+) / calculated av. *m/z* = 1114.83 $[M+3H]^{3+}$, 836.37 $[M+4H]^{4+}$, 669.30 $[M+5H]^{5+}$ for M = $C_{137}H_{211}N_{34}O_{47}S_3Tb$; deconvoluted mass found = 3341.2 / expected mass = 3341.47 (average isotopic composition).

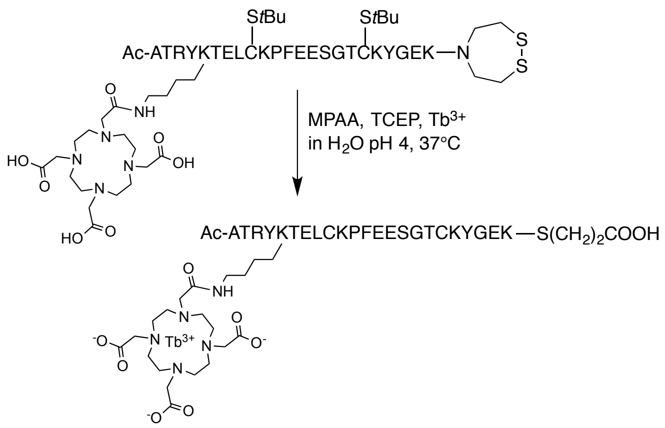

Figure S4. Synthetic pathway for the preparation of the N-terminal segment thioester **1**.

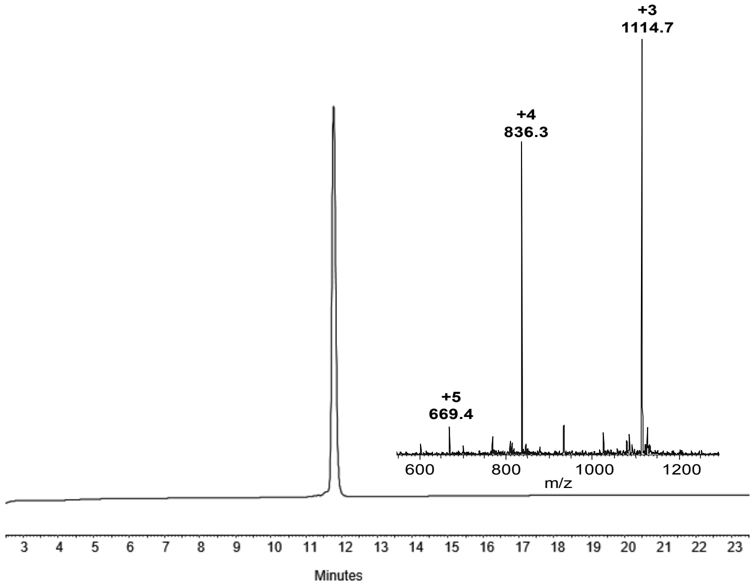

Figure S5. Analytical HPLC chromatogram (λ = 214 nm) and ESI-MS spectrum (positive mode) of the N-terminal segment thioester **1**.

SEA^{off} central segment 2: Peptide elongation was performed as described above on SEA-PS resin (0.1 mmol, 0.16 mmol/g) after attachment of the first amino acid by double manual coupling (30 min) using 10-fold excess of Fmoc-Leu-OH, 9.5-fold excess of HATU and 10-fold excess of DIEA in DMF with pre-activation (5 min) followed by acetylation using (Ac_2O/DIEA/DCM 2:1:17 by vol., 10 mL, 2×5 min).[3] The resin was washed with DMF (2×2 min), DCM (2×2 min) and Et_2O (2×2 min) and dried. Removal of acid-labile side chain protecting groups and cleavage of the peptididyl resin was performed with TFA/H_2O/TIS/thioanisole (92.5/2.5/2.5/2.5 by vol., 10 mL) during 4h. The peptide was then precipitated in ice-cold Et_2O/heptane (1:1 by vol., 100 mL), dissolved in deionized water, and lyophilized. The peptide was dissolved in H_2O/AcOH and treated with a solution of I_2 (200 mM in DMSO) to oxidize the C-terminal SEA^{on} group into SEA^{off} group.[3,5] After 30 s, DTT (65 mM in water, 500 µl) was added to quench the excess of iodine. The oxidized peptide was immediately purified by HPLC to give **2** (39 mg, 12% yield, 0.1 mmol scale). ESI-MS: average *m/z* = 1025.2 (3+), 769.0 (4+), 615.6 (5+) / calculated av. *m/z* = 1025.24 $[M+3H]^{3+}$, 769.19 $[M+4H]^{4+}$, 615.55 $[M+5H]^{5+}$ for M = $C_{138}H_{215}N_{41}O_{31}S_4$; deconvoluted mass found = 3072.9 / expected mass = 3372.71 (average isotopic composition).

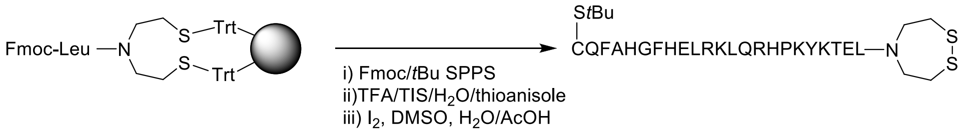

Figure S6. Synthesics pathway for the preparation of the central segment **2**. The S*t*Bu protecting groups is used for the N-terminal cysteine instead of a classical Trityl group to prevent irreversible oxidation of unprotected Cys residues during step (iii), i.e. I_2 oxidation of the SEA group.

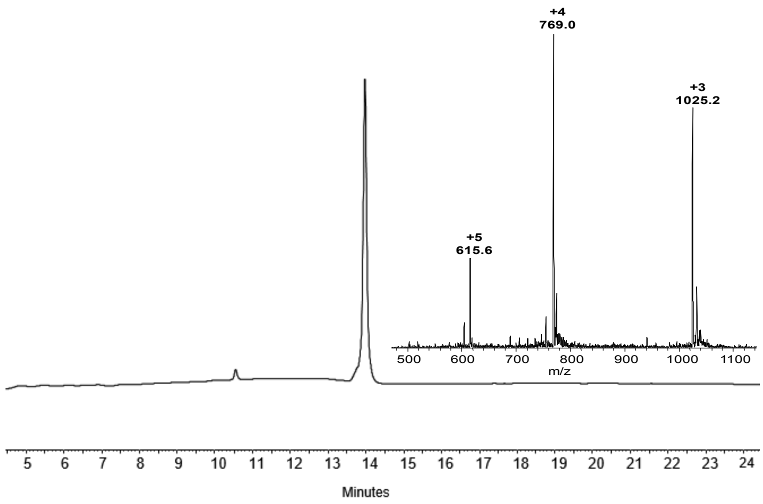

Figure S7. Analytical HPLC chromatogram (λ = 214 nm) and ESI-MS spectrum (positive mode) of the central segment **2**.

C-terminal segment 3: Peptide elongation was performed manually on Rink-PEG-PS resin (Nova PEG Rink Amide, 0.1 mmol, 0.41 mmol/g) after attachment of the first amino acid by single manual coupling (30 min) using 2-fold excess of Fmoc-Leu-OH, 2-fold excess of PyBOP and 6-fold excess of DIEA in DMF followed by acetylation using (Ac_2O/pyridine/DMF 1:2:7 (by vol.), 10 mL, 5 min). Fmoc-Glu(Cs124)-OH (2 eq.) was coupled for 2h using HATU (1.9 eq.) and DIEA (6 eq.) in DMF with 5 min pre-activation and completion of the coupling was controlled by using a TNBS assay. After complete synthesis of the peptide segment, removal of side chain protecting groups and cleavage were performed using TFA/H_2O/TIS/thioanisole (92.5:2.5:2.5:2.5 by vol., 10 mL) for 2 h. The peptide was precipitated in cold Et_2O/heptane (1:1 by vol., 150 mL), centrifugated, dissolved in deionized water, lyophilized and purified by HPLC to give **3** (33 mg, 12% yield, 0.1 mmol scale). ESI-MS: average *m/z* = 922.5 (3+), 692.3 (4+), 554.1 (5+) / calculated av. *m/z* = 922.73 $[M+3H]^{3+}$, 692.30 $[M+4H]^{4+}$, 554.04 $[M+5H]^{5+}$ for M = $C_{126}H_{174}N_{38}O_{28}S_3$); deconvoluted mass found = 2765.2 / expected mass = 2765.17 (average isotopic composition).

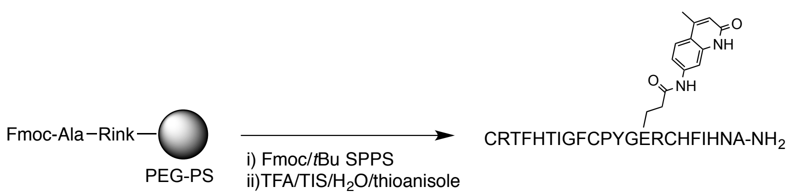

Figure S8. Synthetic pathway for the preparation of the C-terminal segment **3**.

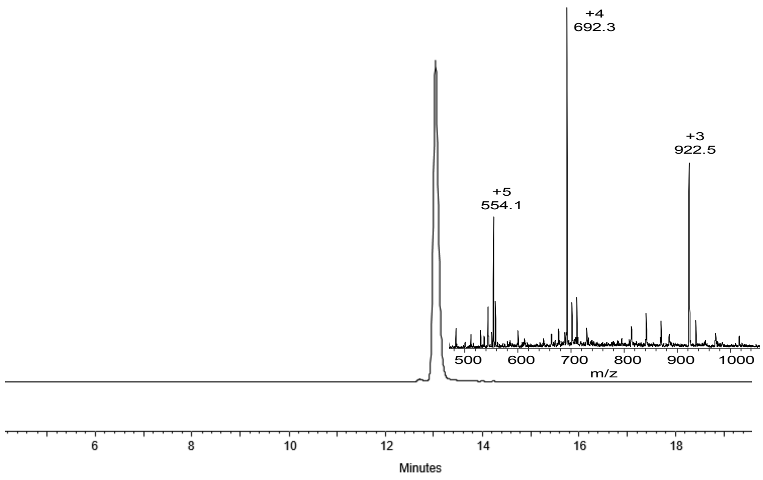

Figure S9. Analytical HPLC chromatogram (λ = 214 nm) and ESI-MS spectrum (positive mode) of the C-terminal segment **3**.

***apo*-LTIS^{Tb}**: A MPAA solution was prepared by dissolving MPAA (34 mg, 0.2 mmol) in a guanidine–HCl (6 M) / sodium phosphate (0.1 M) buffer pH 7.2 (1 mL) and adjusting to pH to 7.1 using aq. NaOH (6 N). A TCEP solution was prepared by dissolving TCEP (143 mg, 0.5 mmol) in a guanidine–HCl (6 M) / sodium phosphate (0.1 M) buffer pH 7.2 (1 mL) and adjusting to pH to 7.1 using aq. NaOH (1 N). All solutions were degassed under argon. Segments **1**, **2** and **3** were assembled by the one-pot methodology described by Melnyk *et al.*[5,6] The N-terminal segment **1** (6 mg, 1.6 µmol) and the central segment **2** (7.3 mg, 1.8 µmol, 1.1 eq) were dissolved in the MPAA solution (233 µL, final peptides concentration 7 mM). The solution was stirred at 37°C but under an argon atmosphere in a plastic reaction tube with a screw cap and the progress of the ligation was monitored by HPLC. After the completion of the ligation of the N-terminal and central segments (*ca.* 24 h as judge by the disappearance of the central segment), the SEA^{off} ligation product was reduced by addition of the TCEP solution (93 µL, final peptide concentration 5 mM). The C-terminal segment **3** (6.7 mg, 1.9 µmol, 1.2 eq.) was then added to the reaction mixture and stirred at 37°C under argon. The formation of the synthetic protein *apo*-LTIS^{Tb} was monitored by HPLC. After 48 h, the reaction was completed. The reaction mixture was diluted with water (2 mL), acidified with 5% aq. TFA (2 mL) and extracted with Et_2O to remove the excess of MPAA. The crude product was directly purified by HPLC to give *apo*-LTIS^{Tb} (7 mg, 40% yield). MALDI-TOF MS (using sinapinic acid as a matrix): observed *m/z* = 8851.2 (average isotopic composition) / calculated *m/z* = 8850.8 $[M+H]^+$ for $C_{390}H_{578}N_{112}O_{104}S_6Tb$.

Expression and purification of the two-domain TTP construct

A construct of TTP, called TTP-2D, that encodes for the two ZF domains of TTP, with the amino acid sequence of: MSRYKTELCRTYSESGRCRYGAKCQFAHGLGELRQANRHPKYKTELCHKFYLQGRCPYGSRCHFI-HNPTEDLAL (aa 107-180) was overexpressed and purified. The expression vector was prepared by ligating the gene fragment of TTP-2D into a pET-15b vector such that the resultant vector eliminated the hexahistidine tag from the coding region.[7] The vector was transformed into BL21-(DE3) competent cells (Novagen) and grown in Luria-Bertani (LB) medium containing 100µg/mL ampicillin at 37˚C until mid-log phase (~OD_{600} of 0.6-0.8) followed by induction of protein expression with 1 mM IPTG (Isopropyl β-D-1-thiogalactopyranoside). At 4 hours post induction, the cells were harvested by centrifugation at 7800 x g for 15mins at 4˚C. Cell pellets were resuspended in 8M urea, 10mM MES pH 6, with EDTA-free protease inhibitor mini-tablet (Roche), lysed by sonication (Fisher Scientific Sonic Dismembrator Model 100) on ice, and centrifuged at 12,100 rpm for 15 minutes at 4˚C to remove cellular debris. The supernatant was applied to a SP-Sepharose column at room

temperature, equilibrated via rocking for 60 minutes, and a step gradient from 0 to 2 M NaCl in 10mM MES, 4M Urea, pH 6 was applied. TTP-2D eluted at 600 mM NaCl. The eluted peptide was then heated in 25 mM DTT at 56˚C for 2 hours to reduce any disulfide bonds. A second purification step was then performed via gradient C18-reverse phase HPLC (Waters 626 bioinert LC). *apo*-TTP-2D eluted at 32% acetonitrile, 68% H_2O. The purified TTP-2D was then transferred to a Coy anaerobic chamber (97% nitrogen/3% hydrogen atmosphere) and the protein was lyophilized using a Savant SpeedVac concentrator. The purity of TTP-2D was assessed via SDS-PAGE and MALDI MS. Zn-TTP-2D was prepared by addition of two equivalents of $ZnCl_2$ to the protein.

Zn-loading of LTIS^{Tb}

Zinc binding to LTIS^{Tb} was monitored by CD and Tb^{3+} luminescence spectroscopies. For CD measurements, *apo*-LTIS^{Tb} was dissolved in a phosphate buffer (20 mM, pH 7.0) containing TCEP (250 mM). The concentration of the protein was determined using measured absorption at 342 nm and reported extinction coefficients for the Cs124 amide chromophore (ε = 10 500 M^{-1} cm^{-1}).[8] A titration was performed in a 0.4 cm path length cuvette using a $ZnCl_2$ solution (1.68 mM) in H_2O. CD spectra were recorded from 195 to 300 nm every 1 nm with 4 s averaging. The CD titration is shown in Figure S10A. For luminescence measurements, the zinc-free probe *apo*-LTIS^{Tb} (0.1 mg) was dissolved in a HEPES buffer (10 mM, pH 7.5) containing DTT (2.5 mM). The concentration of the protein was determined by UV absorption at 341 nm. Aliquots of a $ZnCl_2$ solution (1.68 mM) in H_2O were added to the protein solution and Tb^{3+} luminescence spectra (λ_{ex} = 330 nm) were recorded from 450 to 650 nm in time-gated mode (200 μs delay time) after each Zn^{2+} addition. Figure S10B shows the change in Tb^{3+} luminescence intensity upon Zn^{2+} addition. Both CD and luminescence titrations indicates that the protein binds 2 Zn^{2+} ions. The CD spectrum of *apo*-LTIS^{Tb} is characteristic of a random-coil peptide whereas that of the Zn-loaded LTIS^{Tb} is very similar to the CD spectra reported in the literature for Zn-loaded recombinant TIS11d or TTP RNA binding domains indicating the correct folding of Zn-loaded LTIS^{Tb}.[9,10]

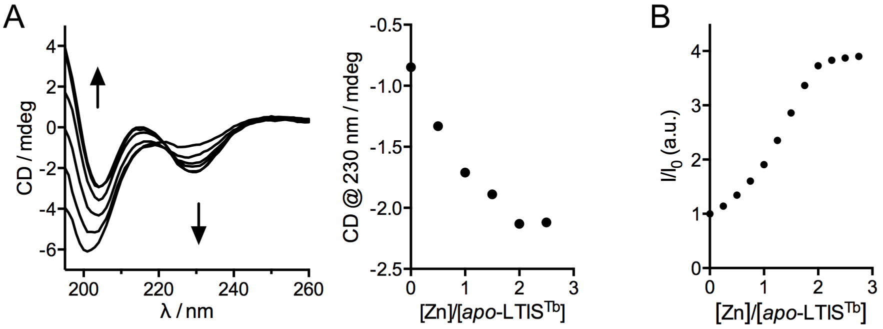

Figure S10. Titration of *apo*-LTIS^{Tb} by Zn^{2+} monitored (A) CD and (B) luminescence spectroscopies. (A) Titration of *apo*-LTIS^{Tb} (3.5 μM) in a phosphate buffer (20 mM, pH 7.0). (B) Titration of LTIS^{Tb} (3.5 μM) in a HEPES buffer (10 mM, pH 7.5). Tb^{3+} emission spectra (λ_{ex} = 330 nm) were recorded in time-gated mode with a 200 μs delay time.

RNA/LTIS^{Tb} interaction: luminescence spectroscopy

^{11}AU binding to LTIS^{Tb}: The titrations of LTIS^{Tb} by the 11-mer RNA 5'-UUUAUUUAUUU-3' (^{11}AU) was performed in a fluorescence cell containing a solution of LTIS^{Tb} (100 nM) in a HEPES buffer (10 mM, pH 7.5, 50 mM NaCl, 2.5 mM DTT, 0.7 mg/mL acetylated-BSA). Aliquots of ^{11}AU (83.4 μM in H_2O) were added successively up to 2.2 equivalents. Tb^{3+} emission spectra (λ_{ex} = 330 nm) were recorded in time-gated mode (delay time = 200 μs) 30 s after each addition of RNA (equilibration is reached within a few seconds). Cs124 fluorescence emission (λ_{ex} = 330 nm) was also recorded during the titration (Figure S11A). A 20% fluorescence

decrease was observed upon ^{11}AU binding. Tb^{3+} luminescence excitation (λ_{em} = 545 nm) spectra recorded in the absence and presence of ^{11}AU demonstrate that Cs124 is the sensitizing antenna (Figure S11C). Tb^{3+} luminescence decays were recorded in the absence and presence of ^{11}AU to determine the Tb^{3+} luminescence lifetime, which remains constant and equal to 1.9 ms (Figure 11D).

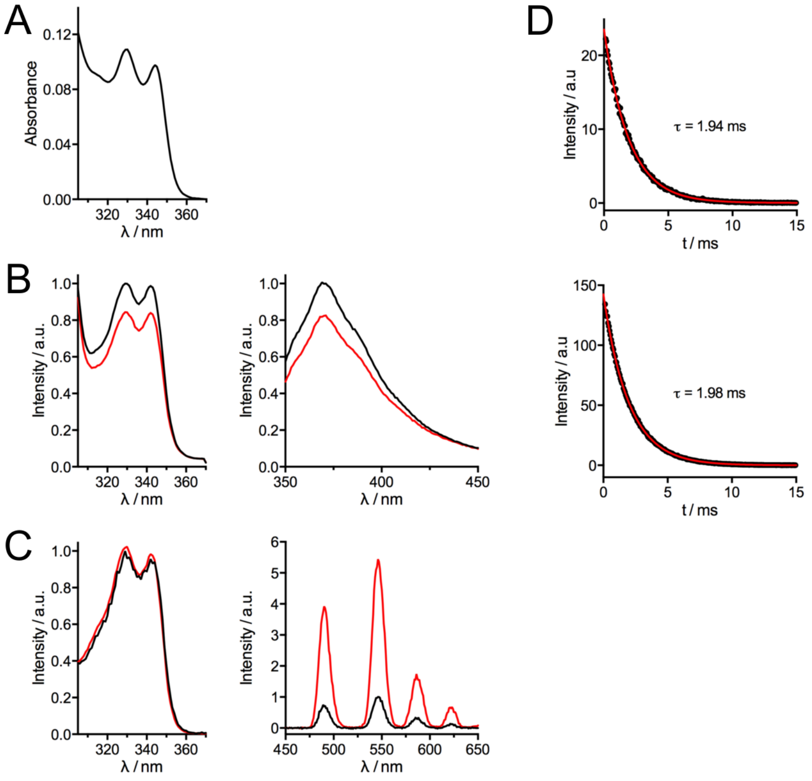

Figure S11. (A) Absorption spectrum of LTIS^{Tb} (9 μM) in a HEPES buffer (10 mM, pH 7.5, 2.5 mM DTT). (B) Fluorescence excitation (λ_{em} = 370 nm, left) and emission (λ_{ex} = 330 nm, right) spectra of LTIS^{Tb} (black) and ^{11}AU·LTIS^{Tb} (red). (C) Tb^{3+} luminescence excitation (λ_{em} = 540 nm, delay = 200 μs, left) and emission (λ_{ex} = 330 nm, delay = 200 μs, right) spectra of LTIS^{Tb} (black) and ^{11}AU·LTIS^{Tb} (red). (D) Tb^{3+} luminescence decay of LTIS^{Tb} (top) and ^{11}AU·LTIS^{Tb} (bottom). Red solid lines correspond to mono-exponential fits, which yielded luminescence lifetimes τ = 1.94 ms and 1.98 ms for LTIS^{Tb} and ^{11}AU·LTIS^{Tb}, respectively.

Titration of ^{11}AU·LTIS^{Tb} by TTP-2D: The affinity of LTIS^{Tb} for the 11-mer RNA ^{11}AU (5'-UUUAUUUAUUU-3') was assessed by competition with the recombinant TTP construct TTP-2D. A fluorescence cuvette containing a solution of LTIS^{Tb} (100 nM) in a HEPES buffer was prepared as described above for titration and 1 equiv. of ^{11}AU was added. Aliquots of TTP-2D were added successively up to 4 equiv. *vs* LTIS^{Tb}. Tb^{3+} luminescence emission spectra (λ_{ex} = 330 nm) were recorded in time-gated mode (delay = 200 μs) after each addition. The titration was fitted using the program SPECFIT,[11] which yielded a value of 0.35 ± 0.03 for the ratio of dissociation constants K_d(LTIS^{Tb})/K_d(TTP-2D). Given a K_d of 16 nM for TTP-2D,[7] this gives a K_d value of 5.6 ± 0.8 nM for the ^{11}AU·LTIS^{Tb} complex.

Selectivity: The binding selectivity of LTIS^{Tb} was investigated by Tb^{3+} luminescence spectroscopy. For this purpose, fluorescence cuvette containing a solution of LTIS^{Tb} (100 nM) in a HEPES buffer were prepared as described above for titrations. A Tb^{3+} luminescence spectrum (λ_{ex} = 330 nm, delay = 200 μs) was recorded. Then, 1 equiv. of various RNA or DNA oligonucleotides (RNAs: 5'-UUUUUUUUUUU-3' (85 μM in H_2O), 5'-UUUGUUUAUUU-3' (82.5 μM in H_2O) and 5'-UUUGUUUGUUU-3' (69.2 μM in H_2O); DNA: 5'-TTTATTTATTT-3' (137 μM in H_2O)) and a Tb^{3+} luminescence spectrum was recorded 30 s after oligonucleotide addition.

References

[1] A. M. Reynolds, B. R. Sculimbrene, B. Imperiali, *Bioconjugate Chem.* **2008**, *19*, 588–591.
[2] B. P. Hudson, M. A. Martinez-Yamout, H. J. Dyson, P. E. Wright, *Nat. Struct. Mol. Biol.* **2004**, *11*, 257–264.
[3] N. Ollivier, L. Raibaut, A. Blanpain, R. Desmet, J. Dheur, R. Mhidia, E. Boll, H. Drobecq, S. L. Pira, O. Melnyk, *J. Pept. Sci.* **2014**, *20*, 92–97.
[4] N. Thieriet, J. Alsina, E. Giralt, F. Guibe, F. Albericio, *Tetrahedron Lett.* **1997**, *38*, 7275–7278.
[5] E. Boll, H. Drobecq, N. Ollivier, A. Blanpain, L. Raibaut, R. Desmet, J. Vicogne, O. Melnyk, *Nat. Protoc.* **2015**, *10*, 269–292.
[6] N. Ollivier, J. Vicogne, A. Vallin, H. Drobecq, R. Desmet, O. El Mahdi, B. Leclercq, G. Goormachtigh, V. Fafeur, O. Melnyk, *Angew. Chem., Int. Ed.* **2012**, *51*, 209–213.
[7] R. C. diTargiani, S. J. Lee, S. Wassink, S. L. J. Michel, *Biochemistry* **2006**, *45*, 13641–13649.
[8] J. Y. Chen, P. R. Selvin, *J. Photochem. Photobiol. A-Chem.* **2000**, *135*, 27–32.
[9] L. M. Deveau, F. Massi, *ACS Chem. Biol.* **2016**, *11*, 435–443.
[10] J. L. Michalek, S. J. Lee, S. L. J. Michel, *J. Inorg. Biochem.* **2012**, *112*, 32–38.
[11] R. Binstead, A. Zuberbühler, *SPECFIT Global Analysis System, Version 3.0*, Spectrum Software Associates, Marlborough, MA, **2000**.
